# A Disintegrin and Metalloprotease 17 in the Cardiovascular and Central Nervous Systems

**DOI:** 10.3389/fphys.2016.00469

**Published:** 2016-10-18

**Authors:** Jiaxi Xu, Snigdha Mukerjee, Cristiane R. A. Silva-Alves, Alynne Carvalho-Galvão, Josiane C. Cruz, Camille M. Balarini, Valdir A. Braga, Eric Lazartigues, Maria S. França-Silva

**Affiliations:** ^1^Department of Pharmacology and Experimental Therapeutics and Cardiovascular Center of Excellence, Louisiana State University Health Sciences CenterNew Orleans, LA, USA; ^2^Centro de Biotecnologia, Universidade Federal da ParaíbaJoão Pessoa, Brazil; ^3^Centro de Ciências da Saúde, Universidade Federal da ParaíbaJoão Pessoa, Brazil

**Keywords:** hypertension, atherosclerosis, shedding, TACE, TNFα, EGFR, ACE2

## Abstract

ADAM17 is a metalloprotease and disintegrin that lodges in the plasmatic membrane of several cell types and is able to cleave a wide variety of cell surface proteins. It is somatically expressed in mammalian organisms and its proteolytic action influences several physiological and pathological processes. This review focuses on the structure of ADAM17, its signaling in the cardiovascular system and its participation in certain disorders involving the heart, blood vessels, and neural regulation of autonomic and cardiovascular modulation.

## Introduction

A Disintegrin and Metalloproteases (ADAM), originally named metalloproteinases disintegrin cystein-rich (MDC), are membrane-anchored cell surface proteins containing both disintegrin and metalloproteinase domains. They belong to the adamalysin protein family in the zinc protease superfamily and combine features of both proteases and cell surface adhesion molecules (Wolfsberg and White, 1996; Seals and Courtneidge, 2003). By 2010, 40 ADAM homologs had been identified in the mammalian genome and 21 ADAM described, among which only 13 were proteolytically active (Edwards et al., 2008). The ADAMs responsible for protein cleavage are called *sheddases*. It is estimated that as much as 10% of the cell surface proteins undergo ectodomain shedding. Members of the ADAM family contribute to various physiological and pathophysiological processes by modulation of molecules like growth factors or cytokines. The prototype of ADAM with sheddase activity is exemplified in ADAM17.

ADAM17 (EC 3.4.24.86), also known as *tumor necrosis factor*-α *converting enzyme* (TACE), is a metallopeptidase which has extensive somatic distribution, being expressed significantly in the heart, vessels, brain, kidney, testicle, placenta, ovary, lung, spleen, and skeletal muscle, with levels varying from embryonic development to adulthood (Black et al., 1997; Patel et al., 1998; Sahin et al., 2004; Dreymueller et al., 2012). ADAM17 was described for the first time by Black et al., as a new member of the family of mammalian adamalysins, to specifically cleave the precursor of tumor necrosis factor α (pro-TNFα; Black et al., 1997; Moss et al., 1997). Until now, ADAM17 was revealed as the major sheddase, of which the substrates cover a diverse variety of membrane-anchored cytokines, cell adhesion molecules, receptors, ligands, and enzymes, such as TNFα, transforming growth factor α (TGFα), L-selectin, and angiotensin-converting enzyme type 2 (ACE2; Kishimoto et al., 1989; Sahin et al., 2004; Weskamp et al., 2004; Lambert et al., 2005; Scheller et al., 2011).

As predicted by the wide variety of ADAM17 substrates, global disruption of the ADAM17 gene *in vivo* results in the death of mice between embryonic day 17.5 and the first day after birth, due to a number of developmental defects from brain, heart, lung, skin, skeletal, and immune system (Peschon et al., 1998; Jackson et al., 2003). In human, a few cases have shown a rare syndrome, in which patients with a homozygous mutation in ADAM17 present severe diarrhea, skin rashes and recurrent sepsis, eventually leading to their early death (Bandsma et al., 2015). On the other hand, due to the structural and functional heterogeneity of ADAM17 substrates, the sheddase is also involved in various pathological processes such as cancer, inflammatory diseases, neurological diseases, cardiac failure, atherosclerosis, diabetes, cardiac hypertrophy, and hypertension (Sandgren et al., 1990; Black et al., 1997; Peschon et al., 1998; Li et al., 2006; Ohtsu et al., 2006b; Zhan et al., 2007; Wang et al., 2009; Scheller et al., 2011; Giricz et al., 2013; Menghini et al., 2013; Xia et al., 2013).

In this review, we summarize the seemingly paradoxical functions of ADAM17 with a particular emphasis on the cardiovascular and central nervous systems (CNS). The structure and structure-based modulation of ADAM17 are also described for better understanding of the various ADAM17 regulatory pathways in different cell types or tissues. Finally, we also discuss the contribution of ADAM17 as a potential therapeutic target in cardiovascular disorder and the neurogenic component of these cardiovascular diseases.

## ADAM17 structure

**Figure 1:**
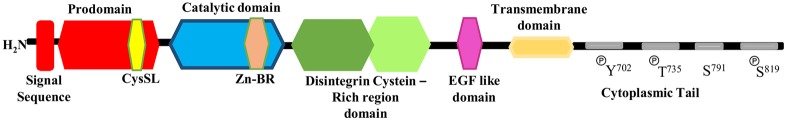
**Schematic representation of the structure of full-length ADAM17**.

ADAM17 has the structural characteristics of disintegrin and metalloproteases proteins that exist predominantly in two forms: as the full-length protein (~100 kDa) and as a mature form lacking the pro-domain (~80 kDa; Gilles et al., 2003). As shown in Figure 1, the full-length protein (or pro-ADAM17) is composed of 824 amino acids and consists of a series of conserved protein domains: an N-terminal signal sequence (aa 1–17), followed by a pro-domain (aa 18–216), in which there is a cysteine switch-like region (CysSL) PKVCGY^186^ (aa 181–188), a catalytic domain (aa 217–474) with a Zn-binding domain region (Zn-BR) (aa 405–417), a disintegrin cysteine-rich domain (aa 480–559), an EGF-like region (aa 571–602), a transmembrane domain (aa 672–694), and a cytoplasmic tail (aa 695–824). Tyr^702^, Thr^735^, Ser^819^ have been shown as cytoplasmic phosphorylation (P) sites, Ser^791^ has been shown as cytoplasmic desphosphorylation site (Patel et al., 1998; Ohtsu et al., 2006a; Gooz, 2010; Xu and Derynck, 2010; Niu et al., 2015).

The pro-domain of ADAM17 includes a cysteine switch box (Galazka et al., 1996), which is an unpaired cysteine residue and play a key role in the pro-domain release prior to ADAM17 activation (Van Wart and Birkedal-Hansen, 1990; Roghani et al., 1999). Normally, the pro-domain of ADAM17 acts as an inhibitor of the protease via linkage of a cysteine switch box (SH-group) to the zinc atom in the active catalytic site. Removal of the pro-domain is a pre-requisite for ADAM17 activation (Reiss and Saftig, 2009). ADAM17 is synthesized and stored in the rough endoplasmic reticulum, the pro-domain removal occurs in a late Golgi compartment, most likely by furin or furin-like pro-protein convertase or by autocatalysis, providing the mature form of ADAM17 (Adrain and Freeman, 2012; Dreymueller et al., 2012). Data from Srour et al., using *in vitro* and *in vivo* cleavage assays, highlight the pro-protein convertases PACE-4, PC5/PC6, PC1, and PC2, as some examples of furin-like enzymes that can directly cleave the ADAM17 protein (Srour et al., 2003). After maturation, ADAM17 translocates to the cell surface to perform proteolytic and non-proteolytic functions (Zhang et al., 2016). Since the pro-domain is highly sensitive to proteolysis, once detached from catalytic domain, it will be degraded rapidly, preventing its re-association with this domain. It has also been suggested that changes in conformation, mediated by the cysteine-rich domain, result in reducing the affinity between pro- and catalytic domains (Milla et al., 1999). Another function of the pro-domain is to chaperone the proper folding of ADAM17 and other ADAM (Loechel et al., 1999; Milla et al., 1999; Anders et al., 2001; Leonard et al., 2005; Li et al., 2009).

The catalytic or metalloproteinase domain starts downstream from the consensus furin cleavage site (RVKRR^215^) and contains a canonical zinc-binding site and a consensus (HEXXH) motif, crucial for the catalytic activity. It mediates shedding of membrane-bound proteins (Maskos et al., 1998; Ohtsu et al., 2006a).

The cysteine-rich domain of ADAM17 includes a disintegrin-like region that can bind to integrins, therefore ADAM17 exhibit both proteolytic and adhesive characteristics (Zhang et al., 2016). Furthermore, Reddy et al. (2000) suggested that the cysteine-rich domain might be involved in recognition of substrates. Following the disintegrin domain, a single transmembrane domain defines the end of the catalytic domain of ADAM17 and then followed by a cytosolic tail that contains consensus sequences for binding to proteins containing Src homology 2 and Src homology 3 domains. Little has been known about the role of this domain in regulating catalytic activity and physiological substrates recognition (Qi et al., 1999).

The role of the cytoplasmic domain in ectodomain shedding still remains controversial. Fan et al. and Gechtman et al. reported that the cytoplasmic domain of ADAM17 is involved in the regulation of ectodomain cleavage in response to intracellular signaling events such as receptor protein tyrosine kinase and mitogen-activated protein kinases (MAPK) activation (Fan and Derynck, 1999; Gechtman et al., 1999). Arguably, soluble ADAM17 forms, lacking trans-membrane and cytoplasmic domain, can cleave trans-membrane proteins or synthetic peptide substrates but not very efficiently (Kahn et al., 1998; Reddy et al., 2000; Fan et al., 2003; Gonzales et al., 2004; Li and Fan, 2004). However, it was soon found that the ADAM17 transmembrane domain proved to play a critical role in appropriately regulating substrate recognition. When the ADAM17 trans-membrane domain was switched with the transmembrane domain of prolactin receptor and platelet-derived growth factor receptor (PDGFR) it strongly inhibit TGFα release, with no change in L-selectin shedding. However, switching the transmembrane domain of TGFα with that of L-selectin actually restored cleavage activity of the transmembrane switched ADAM17 (Li et al., 2007).

## Post-translational regulation of ADAM17

The fact that ADAM17 has a wide diversity of substrates raise the questions about where, when, and how it is activated. Shedding activity led by ADAM17 is sequestered in cholesterol micro-domains within the cellular membrane. Cholesterol is often found to be distributed non-randomly in domains or pools in membranes, therefore these cholesterol-enriched microdomain, also known as “lipid raft,” is thought to be an important micro-environment, in which the signaling pathway can be regulated specifically (Pucadyil and Chattopadhyay, 2006). Depletion of membrane cholesterol by methyl-β-cyclodextrin can induce ADAM17-dependent shedding (Tellier et al., 2006).

It was shown in an early report that ADAM17 cytoplasmic domain was not critical for the phorbol-ester (PMA)-induced TNFα shedding (Reddy et al., 2000). However, considering that PMA is a strong and pleiotropic activator, this cytoplasmic tail could still be necessary for stimulations working through physiological signaling pathways. The cytoplasmic domain of ADAM17, which contains putative phosphorylation sites, is thought to be required for regulation of the metalloprotease activity via several intracellular signals. Now the involvement of MAPK in ADAM17 activation is well-accepted. So far, two phosphorylation sites (Thr^735^, Ser^819^) and one dephosphorylation site (Ser^791^) located on the cytoplasmic tail have been recognized. Mutation or dephosphorylation on Ser^791^ enhance ADAM17 phosphorylation at Thr^735^, which can be decreased by mutation on Ser^819^ (Xu and Derynck, 2010). Replacing Thr^735^ with alanine, which cannot be phosphorylated, resulted in accumulation of non-functional ADAM17 on the cell surface. Both extracellular signal-regulated kinase (ERK) and p38-MAPK pathways activate ADAM17 through Thr^735^ phosphorylation. In mammalian cells, ERK-mediated phosphorylation of ADAM17 at Thr^735^ highlights a key step in inducible ADAM17 protein trafficking and maturation (Soond et al., 2005). Protein kinase C (PKC) used to be considered as an upstream signal of ERK/MAPK pathway, however, it may also have direct role in ADAM17 phosphorylation. A recent study showed that the PKCδ inhibitor, rottlerin, significantly inhibited both constitutive, and high glucose-induced ACE2 shedding, which is mediated by ADAM17, and treatment with PKCδ-targeting siRNA reduced ACE2 shedding by ~20% (Xiao et al., 2016). Other protein kinases, such as protein kinase G (PKG), phosphoinositide-dependent kinase 1 (PDK1), and Polo-like kinase 2 (PLK2), were also involved in ADAM17 phosphorylation in various cell types (Zhang et al., 2006; Chanthaphavong et al., 2012; Schwarz et al., 2014).

PMA can increase ADAM17 activity in multiple ways. An early report suggested that PMA might activate ADAM17 through ROS generation in monocytic cell line (Zhang et al., 2001). Actually, mutation of Thr^735^ had little effect on PMA-stimulated ADAM17 activation. Therefore, other than being an upstream signal of ERK/MAPK pathway, ROS may also have a role in translocating mature ADAM17 from Golgi to the cell surface. In p47^phox^ KO mice, translocation of ADAM17 was partially blunted in cardiomyocytes when the ROS production pathway was blocked (Patel et al., 2014). In addition to ROS, ADAM17-mediated ectodomain shedding can be stimulated by calcium influx in a short term manner. Stimulation with ionomycin (a calcium ionophore) rapidly increased the level of interleukin-6 receptor (IL-6R) shedding in mice, however, it is still unclear whether other ADAM might contribute to this process (Garbers et al., 2011).

Though cytoplasmic phosphorylation is not required for PMA-induced ADAM17 activation, the presence of iRhom2 (inactive rhomboid type 2) was found to be indispensable. Actually, both iRhom1 and iRhom2 are involved in the maturation of ADAM17, and they can compensate each other's function in most of tissues except for the brain and immune system (Maretzky et al., 2013; Li et al., 2015). iRhom1/2 regulate forward trafficking of ADAM17 from endoplasmic reticulum (ER) to Golgi compartment where the pro-domain of ADAM17 can be cleaved (Adrain and Freeman, 2012). However, the underlying mechanism of this phenomenon remains unknown.

## ADAM17-mediated signaling in developmental process

### Notch signaling

ADAM-mediated shedding of Notch receptor is a key step in activating Notch signaling. The Notch receptor and its ligand Delta-l1 are required for neuroepithelial development during embryogenesis (Wakamatsu et al., 1999; De Bellard et al., 2002). The Notch signaling network is an evolutionarily conserved intercellular signaling pathway that regulates interactions between physically adjacent cells. In the brain, Notch1 promotes differentiation of progenitor cells into astroglia and knocking-out the Notch1 gene is lethal at birth (Miller and Gauthier, 2007). Notch receptor is activated by one of five ligands: Jagged1, Jagged2, Delta-l (like) 1, Delta-l3, or Delta-l4, expressed on adjacent cells. Following ligand binding, Notch receptor is cleaved by ADAM17 at site 2 (S2) and then by γ-secretase at site 3 (S3) (Kopan and Ilagan, 2009), and so is the ectodomain release of Delta and Jagged (Murthy et al., 2012; Groot et al., 2013). Both murine Notch1 and Notch2 require ADAM17 instead of ADAM10 during ligand-independent activation, which is resisted by the human Notch2 (Habets et al., 2015). Since there are major cardiovascular defects in the ADAM17 global knockout mice, which failed to survive postnatal, ADAM17 would also play a role in vascular physiology. In the adult epidermis, ADAM17 permits tonic Notch activation to regulate epithelial cytokine production and maintain barrier immunity. During cerebral angiogenesis, Notch signaling is initiated by receptor-ligand recognition between adjacent cells (Serra et al., 2015). Overexpression of ADAM17 can promote angiogenesis by increasing blood vessel sprouting and pericytes number during brain micro vessel development (Lin et al., 2011).

### EGFR signaling

Within the cardiovascular system, Shi et al. (2003) demonstrated that ADAM17 possibly regulates cardiomyocyte proliferation during the late fetal stage of cardiac development via an epithelial growth factor receptor (EGFR)-mediated pathway. It was showing in the ADAM17 global knockout mice, a remarkably enlarged heart with increased myocardial trabeculation and reduced cell compaction (Shi et al., 2003). It has also been reported that mice lacking functional ADAM17 suffer from several cardiac abnormalities in valvulogenesis, such as thickened, deformed semilunar, and atrioventricular valves (Jackson et al., 2003). Importantly, these lethal cardiac abnormalities resulted from insufficient EGFR activation, which were due to the lack of ADAM17 (Blobel, 2005). The up-regulation of EGFR by ADAM17 stems from the critical ability of this metalloprotease to cleave multiple EGFR ligands, such as EGF itself, TGFα; epiregulin; heparin binding EGF-like growth factor (HB-EGF), and neuregulins β1 and β2 (Peschon et al., 1998; Merlos-Suárez et al., 2001; Hinkle et al., 2004; Sahin et al., 2004; Horiuchi et al., 2005; Saftig and Reiss, 2011). Neuregulins β1/2 are considered to be critical to cardiac development (Meyer and Birchmeier, 1995; Meyer et al., 1997; Britsch et al., 1998). Arguing against potential compensatory or redundant functions of other members from the ADAM family, quadruple knockout mice lacking ADAM 9, 12, 15, and 17 do not have a more severe phenotype than mice lacking only ADAM17 (Sahin et al., 2004).

In addition to the defects on cardiac development, removal of ADAM17 from subcortical white matter (SCWM) of postnatal mice (90 days) led to abnormalities in Schwann myelination causing impaired motor behaviors (Palazuelos et al., 2014). ADAM17-induced shedding of EGFR ligands, HB-EGF and TGFα, promotes the expansion of oligodendrocyte progenitor cells during the critical periods of Schwann myelination. However, neuregulin-1 cleavage mediated by ADAM17 can negatively regulate myelination in the peripheral nervous system. Down-regulation of ADAM17 expression or its inactivation in motor neurons can lead to abnormal myelination (La Marca et al., 2011).

## ADAM17-mediated cytokines and adhesion molecules signaling

### In the heart

Soluble TNFα, a potent cytokine, is converted from pro-TNFα through ADAM17-mediated cleavage in various cell types. In heart, TNFα is crucially involved in the genesis and progression of several cardiovascular processes. Satoh et al. (1999) have shown that myocardial ADAM17 and TNFα expression in both mRNA and protein levels are increased in humans with dilated cardiomyopathy (Satoh et al., 1999), establishing a positive correlation between ADAM17 and TNFα in cardiovascular disorder. They later showed that increased expression of TNFα and ADAM17 had important implications in advanced cardiac dysfunction in myocarditis. In the myocardium, TNFα contributes to reversible and irreversible ischemia/reperfusion injury, post myocardial infarction remodeling, and heart failure development. Simultaneously, TNFα is also involved in cardio-protective processes in ischemic conditioning. The harmful and beneficial roles of TNFα appear to be dose- and time-dependent and in part related to the activation of specific receptor subtypes (Kleinbongard et al., 2010).

One possible pathway to activate TNFα in the myocardium is through EGFR and many studies have shown that ADAM17 is the main contributor to EGFR transactivation in cardiovascular system (Ohtsu et al., 2006b; Takayanagi et al., 2015). Furthermore, Küper et al. (2007) were the first to suggest a co-dependency between ADAM17-mediated up-regulation of EGFR and TNFα. They observed that within renal collecting duct cells LPS induces EGFR activation via TLR4/ADAM17 (Küper et al., 2007). Based on this study, Sun et al. (2015) have found that this promotes myocardial TNFα production and cardiac failure in endotoxemic mice (Sun et al., 2015). Another TNF-α activation pathway in cardiac cells by ADAM17 is through the non-receptor tyrosine kinase, Src. Src mediates ADAM17 activation in mechanically stretched rat cardiomyocytes by phosphorylating the Tyr^702^ residue within the intracellular tail of ADAM17, leading to activation of p38 MAPK and thus TNF-α receptor activation (Niu et al., 2015).

The association between ADAM17 and cardiac function is not limited to local effects in the heart. Akatsu et al. (2003) demonstrated an increased expression of TNFα and ADAM17 in circulating leukocytes of patients with acute myocardial infarction (AMI) associated with higher plasma TNFα levels when compared with healthy control patients (Akatsu et al., 2003). Shimoda et al. (2005) confirmed the positive correlation between ADAM17 and TNFα levels in AMI (Shimoda et al., 2005). In addition, they observed that these levels were higher in peripheral blood mononuclear cells from AMI patients with in-hospital complications, such as pump failure, malignant ventricular arrhythmia, recurrent myocardial infarction, and cardiac death. Moreover, ADAM17 expression and TNFα levels were found to be increased in peripheral blood mononuclear cells, especially from individuals with advanced congestive heart failure (Satoh et al., 2004).

### In the vasculature

ADAM17 expression is found throughout endothelial cells, smooth muscle cells, fibroblasts, and leukocytes (Dreymueller et al., 2012). The consequences of shedding events for vascular biology depend on the type of substrate shed. This can result in pro- or anti-inflammatory effects depending on the nature of the transmembrane protein cleaved, the generation of soluble receptor agonists or antagonists, modulation of cellular responsiveness, modulation of adhesive properties, and formation of transcription factors (Canault et al., 2006). Interestingly, ADAM17 has been described to be overexpressed in ruptured coronary plaques from infarcted patients and atherosclerotic plaques from apolipoprotein E knockout mice (apoE^−/−^, an important experimental model of atherosclerosis; Canault et al., 2006; Satoh et al., 2008), revealing its fundamental role in this vascular disease.

ADAM17 activity is correlated to adverse clinical outcomes in acute coronary atherosclerosis (Satoh et al., 2008; Gutiérrez-López et al., 2011; Rizza et al., 2015). An important group of substrates for ADAM17 includes adhesion molecules such as ICAM-1 (intercellular adhesion molecule-1), VCAM-1 (vascular cell adhesion molecule-1), L-selectin, and others. These adhesion molecules are involved in leukocyte migration through the vessel wall which is one of the first steps leading to atheroma formation (Gutiérrez-López et al., 2011; Freitas Lima et al., 2015). Shedding of cell adhesion molecules weakens cell–cell interactions and reduces adhesiveness of leukocytes (Arribas and Esselens, 2009; Dreymueller et al., 2012). It has been reported that ADAM17 null leukocytes present increase L-selectin levels (Tang et al., 2011). Interestingly, Zhang et al. demonstrated that nitric oxide (NO) is a critical factor involved in ADAM17 activation due to nitrosilation of thiol group in cysteine residues in the inhibitory pro-domain of ADAM17. This might explain the increased adhesion of leukocytes in dysfunctional endothelium (Zhang et al., 2000), which presents reduced NO bioavailability (Balarini et al., 2013).

### In the central nervous system

Kärkkäinen et al. (2000) was the first to demonstrate the expression of ADAM17 mRNAs in adult mouse and rat brains, using *in situ* hybridization (Kärkkäinen et al., 2000). ADAM17 showed a restricted pattern of distribution in the telencephalon and diencephalon. Within the mesencephalon, ADAM17 mRNA was detected in the hippocampus along with the inferior colliculus. In addition, ADAM17 mRNA was also detected in the cerebellar cortex, pontine nuclei, and cerebral cortex. According to these data, Hurtado et al. (2001) demonstrated constitutive expression of ADAM17 protein in rat brain (Hurtado et al., 2001). Aspects related to cell location and origin of ADAM17 have been clarified in later studies.

Goddard et al. (2001) in an immunohistochemical study showed that ADAM17 is expressed in astrocytes and endothelial cells from healthy adult human brain tissue and may have a role in normal brain function (Goddard et al., 2001). Hurtado et al. (2002) also found ADAM17 protein expression in astrocytes as well as rat microglial cells (Hurtado et al., 2002). Skovronsky et al. (2001), using Western Blots and immunolabeling approaches, found that ADAM17 was present in neurons of the hippocampus, cortex and cerebellum, and was slightly detectable in astrocytes, oligodendrocytes, and microglial cells (Skovronsky et al., 2001). Since ADAM17 is expressed in different cell types in the CNS and promotes ectodomain shedding of several molecules, this enzyme would participate in several cellular events.

Increased plasma ADAM17 activity was found in patients with mild cognitive impairment. Within the brain, ADAM17 can work as an α-secretase and cleave the amyloid precursor protein (APP) into soluble APPα fragment (sAPPα; Skovronsky et al., 2001; Asai et al., 2003). This shedding of APP is able to reduce the generation of neurotoxic amyloid β (Aβ) peptide in a competitive way. It is believed to be a possible neural protection and repair mechanism. On the contrary, ADAM17 is also a neuro-inflammatory target which responds to ischemia and other stress factors (Romera et al., 2004; Munhoz et al., 2008). Some of the subsequently increased inflammatory factors, like TNFα, can activate glial cells and be harmful to homeostasis within the brain (Suzumura, 2013). From this point of view, activation of ADAM17 can be neurotoxic. As described before, ADAM17 was initially reported to be responsible for the proteolytic activation of the membrane precursor of TNFα, which is critically involved in inflammation. The pro-inflammatory activity of TNFα is predominantly mediated by TNFα receptor type 1 (TNFR1) and to some extent by TNF receptor type 2 (TNFR2), both of which have been found to undergo shedding via ADAM17 (Chanthaphavong et al., 2012). Wang et al. (2003) had linked the shedding of TNF receptors to neutralization of soluble TNFα-induced actions. Either ADAM17 expression or activity have found to be altered in neuro-inflammatory conditions, in which, the level of TNFα is increased, such as stroke, multiple sclerosis, or traumatic brain injury (Romera et al., 2004; Plumb et al., 2006). Conditional deletion of the ADAM17 gene or inhibition of the protein, effectively blocks LPS-induced TNFα release and systemic inflammation in mice (Zhang et al., 2004). This demonstrates that ADAM17-mediated shedding is a critical trigger for pro-inflammatory signaling of TNFα. In addition, BB1101, a blocker of ADAM17, was shown to be effective in reducing TNFα levels in stressed animal models (Madrigal et al., 2002). Interestingly, the specific antagonist of NMDA receptor, MK-801, can, not only decrease stress-induced activity and expression of ADAM17, but also its constitutive expression, as well as TNFα levels (Madrigal et al., 2002). Therefore, in the CNS, excitation or stimulations targeting glutamatergic neurons may cause up-regulation of ADAM17 activity and will contribute to neuro-inflammation eventually. The mechanism of this neuro-excitation-induced ADAM17 activation is thought to be mediated via nuclear factor-κ B (NFκB). Stress-induced neuro-inflammation activates NFκB in the hippocampus as soon as 4 h after the onset of stress in rats (Madrigal et al., 2006). The implication of ADAM17 and TNFα in stress-induced activation of NFκB was confirmed after finding that decreased TNFα is mediated by hepatocyte growth factor-like protein-induced decrease in NFκB activation and increased by the NFκB inhibitory protein, IκB (Nikolaidis et al., 2010).

Besides TNFα, inflammation is also characterized by elevated levels of interleukin-6 (IL-6). ADAM17 is the main sheddase for IL-6 receptor (IL-6R) to induce IL-6 trans-signaling (Pruessmeyer and Ludwig, 2009). Released IL-6R is capable of binding IL-6 and the formed receptor-ligand complex then stimulates gp130 on the cell surface, even in the absence of transmembrane IL-6R (Scheller et al., 2011). This pathway is thought to play an important role in chronic inflammatory processes. In immune cells, like neutrophils, ADAM17 and secondary shedding of IL-6R can be activated by apoptosis (Chalaris et al., 2007).

In the nervous system, expression of cell adhesion molecules (CAMs) can be up-regulated by glia-secreted cytokines. Shedding of the CAMs, such as CX3CL1 (fractalkine), L-selectin, VCAM-1, and JAM-A (junctional adhesion molecule-A) are induced by activation of ADAM10 or ADAM17 (Kalus et al., 2006; Pruessmeyer and Ludwig, 2009). In the periphery, CX3CL1 guides leukocytes to the site of inflammation. Within 4–6 h after onset of ischemia, circulating leukocytes adhere to vessel walls and migrate into the brain with subsequent release of additional pro-inflammatory mediators. The ADAM17-related adhesion procedure is critical during the process of inflammation (Lakhan et al., 2009). On the other hand, CX3CL1 can be secreted from damaged neurons and then activate microglia to rescue neurons by up-regulating phagocytosis of toxicants or damaged debris and production of anti-oxidant enzymes, like superoxide dismutase (Vernon and Tang, 2013; Limatola and Ransohoff, 2014). Furthermore, ADAM17-mediated shedding is important to neuronal outgrowth in the developmental process. ADAM17 regulates L1CAM (L1-cell adhesion molecule)-dependent neuronal cell adhesion, cell migration, as well as neurite outgrowth (Mechtersheimer et al., 2001; Maretzky et al., 2005). L1CAM has been observed within late embryonic/early postnatal cortical neurons and fibers in the corpus callosum and corticospinal tract (Fujimori et al., 2000; Jakovcevski et al., 2013). In mice embryonic cortex, knocking-down ADAM17 could affect L1-involed neuronal intermediate progenitor cells multipolar exit and migration (Li et al., 2013).

## ADAM17-mediated growth factors signaling

### In vascular remodeling and formation of atherosclerotic plaques

ADAM17 is expressed in atherosclerosis-prone sites, however it is not clear whether this expression is due to an up-regulation of enzyme expression or due to its expression by newly infiltrated vascular smooth muscle cells (VSMCs) and leukocytes (Canault et al., 2006). It is well-established that Ang-II can induce VSMC hypertrophy, proliferation and migration, which are important steps toward atheroma formation (Freitas Lima et al., 2015). Ang-II can induce ADAM17 protein expression in the vasculature and increase its activity by tyrosine phosphorylation (Elliott et al., 2013; Obama et al., 2015). ADAM17-induced EGFR transactivation is considered a major contributor in Ang-II-induced vascular remodeling. It was reported that Ang-II induces expression of fibronectin and transforming growth factor-β (TGFβ) through downstream signaling of EGFR transactivation and ER stress. This occurs via a signaling mechanism involving ADAM17-mediated shedding which is independent of hypertension (Moriguchi et al., 1999; Takayanagi et al., 2015).

In atherosclerotic plaques, neovascularization occurs as a physiological response to increased oxygen demand which causes adverse effects by facilitating inflammatory influx and favoring the conditions that destabilize the plaque. Thus, inhibition of neovascularization results in limited atherosclerotic lesion (van Hinsbergh et al., 2015). ADAM17 positively regulates angiogenesis by inhibiting the expression of the anti-angiogenic factor thrombospondin 1, presenting a positive role in pathological angiogenesis (Caolo et al., 2015). ADAM17 is also critical for EGFR signaling due to the proteolytic release of several ligands of EGFR in the vessels, such as HB-EGF. Weskamp et al. demonstrated that inactivation of ADAM17 in endothelial cells reduced pathological neovascularization whereas its inactivation in smooth muscle cells revealed no evident effect (Weskamp et al., 2010). The underlying mechanism is probably EGFR signaling stimulated by EGFR ligands released by ADAM17 from endothelial cells. Selective inhibition of ADAM17 could be beneficial for the treatment of diseases implied in pathological neovascularization (Weskamp et al., 2010), such as atherosclerosis. Vascular endothelial growth factor (VEGF-A) and its receptor (VEGFR) are also critical for regulating angiogenesis in physiological and pathological conditions. VEGFR type 2 is a tyrosine kinase receptor which is considered the principal coordinator of adult angiogenesis (Swendeman et al., 2008). This receptor can be shed from cells by ADAM17. VEGF-A/VEGFR2 were shown to stimulate ADAM17, resulting in shedding of VEGFR2 and other ADAM17 substrates (Swendeman et al., 2008). This could limit angiogenesis process and reveal a potential novel target for treatment of pathological neovascularization associated to atherosclerosis. It is important to highlight that ADAM17 over-expression or inhibition could either increase or decrease neovascularization as it also presents pro- or anti-inflammatory functions.

Plaque rupture and subsequent thrombotic complications are adverse events associated with atherosclerosis (Freitas Lima et al., 2015). Local over-activity of ADAM17 may weaken atherosclerotic plaques, causing its rupture. In this context, Rizza et al. suggested that measuring ADAM17 activity may predict major cardiovascular events in subjects with established atherosclerosis (Rizza et al., 2015). Although ADAM17 activity was evaluated in an indirect manner through the measurement of its substrates in circulation, authors suggest that it is reasonable to assume that the increase in these molecules is related to increased ADAM17 activity in inflammatory sites. In addition, it was observed that unsaturated fatty acids in LDL particles are also involved in ADAM17 activation in the endothelial layer, due to increase in membrane fluidity, creating a link between endothelial dysfunction/atherosclerosis and increase in ADAM17 substrates in patients at risk (Reiss et al., 2011; Menghini et al., 2013).

### In brain injury and tumor

Over the years, an important role for ADAM17 in neural injury and degeneration has gradually become clear. Via shedding of TGFα, ADAM17 can work as the constitutive sheddase of epiregulin and amphiregulin, both members of the epidermal growth factor (EGF) family playing important roles in the regulation of cell growth, proliferation, and survival (Freimann et al., 2004; West et al., 2008). Recombination of epiregulin and amphiregulin proteins can effectively inhibit endoplasmic reticulum stress and the subsequent induction of neuronal cell death. Therefore, up-regulation of epiregulin and amphiregulin in glial cells may have neuro-protective effects and provide a potential therapy for brain injury (Zhan et al., 2015). However, there is evidence suggesting an association between increased expression of ADAM17 and various types of cancer. EGFR binding with ligands after ADAM17-induced shedding can subsequently activate MEK/ERK and PI3K/Akt pathways, which contribute to the invasiveness and other malignant phenotypes of tumors. Glioma is the most common malignant intrinsic primary brain tumor. By up-regulating the ligands of EGFR, ADAM17 can promote glioma cells malignant phenotype by increased proliferation, invasion, and angiogenesis. In glioma cells and glioma-bearing nude mice, targeting ADAM17 with TAPI-2 (an ADAM17 inhibitor) or siRNA, can significantly attenuate tumor growth and invasiveness, compared to their untreated counterparts (Zheng et al., 2012). Further, it has been shown that beyond the EGFR pathway, TGFβ (transforming growth factor-β) also plays a key role in the regulation of glioma formation and progression (Lu et al., 2011). Interestingly, TGFβ, which can rapidly induce ADAM17 phosphorylation, is actually an upstream signal of ADAM17 (Wang et al., 2008). However, the mechanism of TGFβ-induced tumor formation and progression is not simply mediated by activation of the downstream EGFR pathway. Mu et al. found that TGFβ utilized TRAF6 (TNFR-associated factor 6), PKC ζ and ADAM17 to promote the formation of the TβRI (serine/threonine kinase receptor I) intracellular domain, which could be translocated to the nucleus, where it promotes tumor invasion by induction of *Snail* and MMP2 (matrix metalloproteinase-2; Mu et al., 2011).

## ADAM17-mediated ACE2 shedding

### In the cardiac renin angiotensin-system (RAS)

Overactivity of the RAS contributes to the development and maintenance of cardiac hypertrophy in experimental models and humans. Whilst the primary physiological role of angiotensin-converting enzyme (ACE) in the RAS is to hydrolyze Ang-I into the potent vasoconstrictor Ang-II, ACE2 is able to cleave Ang-II to produce Ang-(1–7), a peptide which has opposing effects (Xia and Lazartigues, 2008). Therefore, ACE2 is an important regulator within the RAS. Cardiac hypertrophy and impaired contractility are associated with decreased ACE2, whereas ACE2 over-expression protects the heart from Ang II-mediated cardiac hypertrophy and myocardial fibrosis (Bodiga et al., 2011; Patel et al., 2012). Interestingly, neuronal overexpression of ACE2 has also therapeutic effects on Ang-II-induced cardiac hypertrophy (Feng et al., 2012), confirming the pivotal role of ACE2 within the brain RAS and in the central regulation of cardiovascular function. The compensatory role of ACE2 is compromised, as it is a subject of ADAM17-mediated shedding. The ACE2 ectodomain is cleaved from the cell membrane by ADAM17 and released into the extracellular milieu (Lambert et al., 2005). This further fuels the involvement of ADAM17 in cardiac hypertrophy, as has been shown in both animal and human studies. For example, systemic treatment of ADAM17-targeting siRNA for 30 days effectively stopped the progression of agonist-induced cardiac hypertrophy and fibrosis in adult spontaneously hypertensive rats and mice following Ang-II infusion (Wang et al., 2009).

Similarly, Patel et al. (2014) found that in mice infused with Ang-II, there was an angiotensin receptors type I (AT_1_R)-mediated increase in myocardial ADAM17 expression and activity. Membrane translocation of ADAM17 was linked to a substantial reduction in myocardial ACE2 protein and activity with a corresponding increase in plasma ACE2 activity, suggesting that Ang-II-induced ACE2 shedding was mediated by ADAM17. Reactive oxygen species seem to play a key role since p47^phox^ knockout mice were resistant to Ang-II-induced ADAM17 activation with preservation of myocardial ACE2 and attenuated Ang-II-mediated cardiac dysfunction and hypertrophy (Patel et al., 2014).

### In the brain RAS

Lautrette et al. (2005) demonstrated that peripheral inhibition of ADAM17 with TAPI-2 does not decrease systolic blood pressure in Ang II-infused mice, suggesting that cardiac ADAM17 participates in the development of cardiac hypertrophy, but does not play a primary role in the regulation of blood pressure in this model (Lautrette et al., 2005). However, our group has recently demonstrated that ADAM17 in the brain actually contributes to the development of neurogenic hypertension (Xia et al., 2013). Similar to the heart, overactivity of the brain RAS is a major contributor to the development and maintenance of neurogenic hypertension (Itaya et al., 1986; Davisson et al., 1998). Deoxycorticosterone acetate (DOCA)-salt treatment, a well-characterized model for neurogenic hypertension (Yemane et al., 2010), led to enhanced ADAM17 expression and activity. This ultimately reduced the expression and activity of ACE2 in the mouse hypothalamus (Xia et al., 2013). In addition, the treatment with tempol or α-lipoic acid, blunted ADAM17 activity and preserved ACE2 compensatory effects, suggesting a possible role of oxidative stress in the up-regulation of ADAM17 during DOCA-salt hypertension (de Queiroz et al., 2015).

The activation of ADAM17 by DOCA-salt treatment could be dependent on AT_1_R. Following AT_1_R activation, downstream signaling pathways involving ROS, PKC, MAPK, and calcium (Ca^2+^) might not only up-regulate the activity of ADAM17 but also interfere with the protective effects of ACE2 by interacting with other molecules. In ACE2-expressed HEK cells, calmodulin (CaM) interacts with ACE2. Inhibiting CaM increased the release of the ACE2 ectodomain in a dose- and time-dependent manner (Lambert et al., 2008). This association between CaM and ACE2 has also been reported in the CNS. In mice with DOCA-salt hypertension, interaction between ACE2 and CaM was found to be decreased (Xia et al., 2013), covceivably because of an increase in intracellular [Ca^2+^]. The structure of CaM can be expanded by its specific Ca^2+^ binding sites, which possibly contributes to the dissociation of CaM from the cytoplasmic site of ACE2. This might render ACE2 vulnerable to the shedding activity of ADAM17.

The balance between the classic RAS and the ACE2-related compensatory axis is important to the maintenance of a normal blood pressure as well as central regulation of autonomic function. In mouse basolateral amygdala, overexpression of ACE2 significantly increased the frequency of spontaneous inhibitory postsynaptic currents (indicative of presynaptic release of GABA) from pyramidal neurons. This effect was eliminated by central administration of a Mas receptor (MasR) antagonist, suggesting a possible role of ACE2 in GABA neurotransmission via MasR activation (Wang et al., 2016). Accordingly, ADAM17 can contribute to increased sympathetic outflow by down-regulating ACE2 activity, which in turn exacerbates the contribution of classic RAS. In mice with ACE2 deletion, we observed an increase of baseline blood pressure, which could reach hypertensive levels with age (Xia et al., 2011).

Moreover, ADAM17 can control the transcription of matrix metalloproteinase-2 (MMP-2), which in turn mediated angiotensin-II (Ang-II)-induced hypertension in mice independently of cardiac hypertrophy or fibrosis, showing that the effects of ADAM17 in the cardiovascular system may be connected to other metalloproteinases (Odenbach et al., 2011).

**Figure 2:**
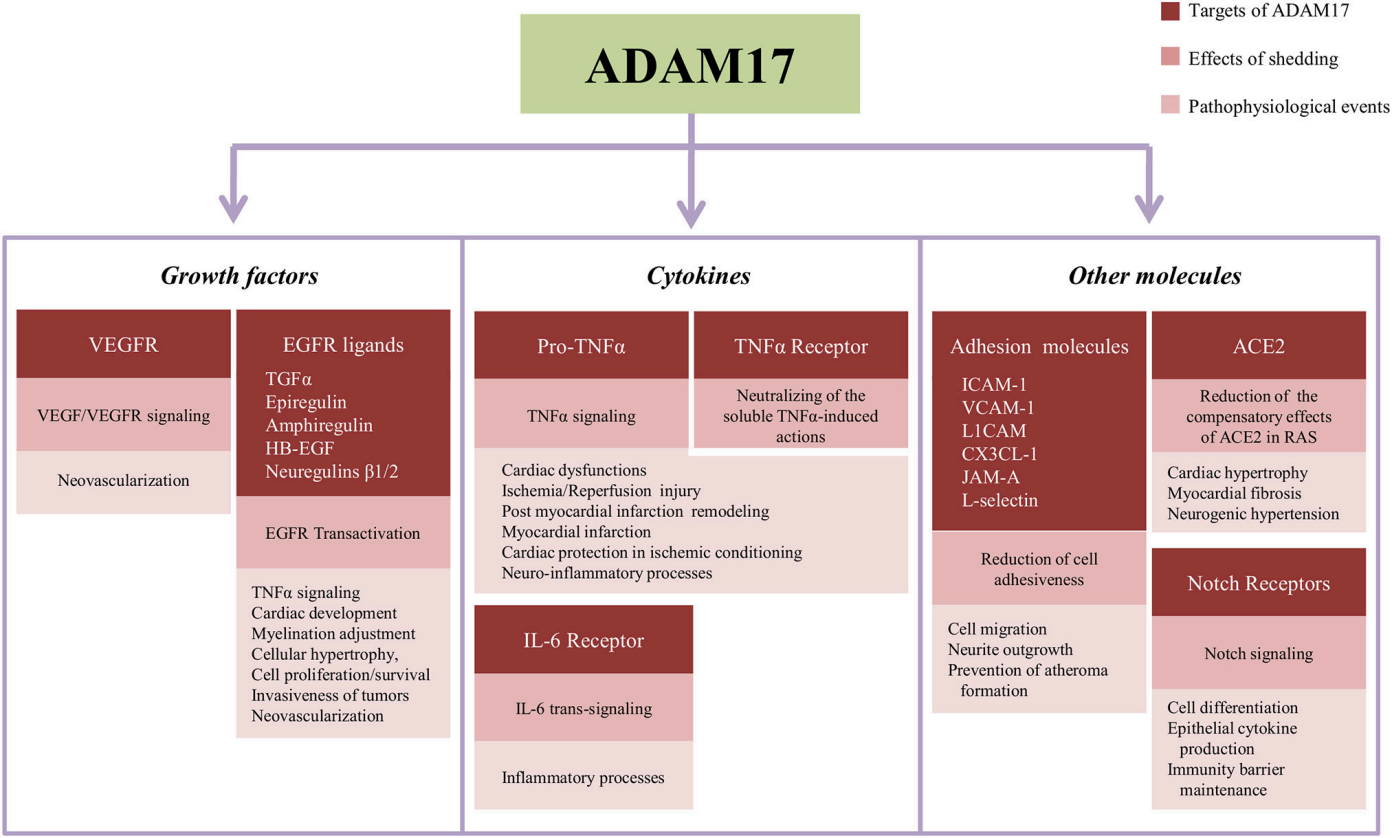
**Summary of targets of the shedding induced by ADAM17, subsequent effects in the cell signaling and events influenced by proteolytical cleavage in the cardiovascular and nervous systems**. ACE2, angiotensin-converting enzyme type 2; CX3CL1, fractalkine; EGFR, epithelial growth factor receptor; HB-EGF, EGF-like growth factor; ICAM-1, intercellular adhesion molecule-1; IL-6, interleukin-6; JAM-A, junctional adhesion molecule-A; L1CAM, L1-cell adhesion molecule; RAS, Renin Angiotensin-System; TGFα, transforming growth factor α; TNFα, tumor necrosis factor α; VCAM-1, vascular cell adhesion molecule-1; VEGF, vascular endothelial growth factor; VEGFR, vascular endothelial growth factor receptor.

The Figure 2 summarizes the main targets of ADAM17-induced shedding described in the text, the downstream events of their proteolytic cleavage and pathological and physiological processes influenced by ADAM17-dependent shedding.

## ADAM17 as a therapeutic target

### A brief introduction of endogenous ADAM17 inhibitor

ADAM share characteristics with the wider family of matrix metalloproteinases (MMP), which are regulated by a group of endogenous inhibitors. Among them, tissue inhibitors of metalloproteinase 3 (TIMP3) not only blocks the activity of MMP but also inhibits the ectodomain shedding induced by ADAM17 (Amour et al., 1998). Both ADAM17 and TIMP3 have high abundance in the heart, kidney and brain. Mice with TIMP3 deficiency show significant increase in ADAM17 activity and soluble TNF-α abundance (Federici et al., 2005; Guinea-Viniegra et al., 2009). This is believed to be responsible for Ang-II-induced vascular inflammation and remodeling (Basu et al., 2012, 2013). Zheng et al. (2016) demonstrated that enhanced ADAM17 expression with decreased TIMP3 and increased TNF-α expression, within 1 week after AMI, are associated with cardiac remodeling (Zheng et al., 2016). These data confirm that ADAM17 is an important regulator of TNF-α maturation and may be a potential target for the inhibition of cellular TNF-α production in cardiovascular disorders. In addition, it was demonstrated that an imbalance between ADAM17 and TIMP3 is characteristic of unstable carotid plaques (Menghini et al., 2013; Rizza et al., 2015). On the other hand, treatment with soluble TIMP3 can impart a neuro-protective effect and enhance neurite out-growth both *in vitro* and in animals with brain-injury, *in vivo* by activating the Akt-mTORC1 signaling pathway (Gibb et al., 2015).

In normal baseline conditions, the cytoplasmic tail of ADAM17 supports cell surface homo-dimerization. The ADAM17 homodimers associate with TIMP3, which silences the shedding activity of ADAM17. Phosphorylation on the cytoplasmic domain of ADAM17 disrupts the homodimers into monomers. This relieves TIMP3 association from ADAM17 enabling it to increase proteolysis of its substrates and hence shifting away from normal baseline condition (Xu et al., 2012).

### Existing knockout models

Because of its essential role in normal fetal development, disruption of the *ADAM17* gene leads to perinatal lethality with opened eyes, defects in aortic, pulmonic, and tricuspid heart valves (Li et al., 2015). Mice with a targeted deletion of exon11 that encodes the catalytic active site of the metalloprotease domain (ADAM17^ΔZn/ΔZn^), resulting in a lack of enzymatic activity, also display substantial perinatal lethality within 2 weeks. Though one study reported that some ADAM17^ΔZn/ΔZn^ null mice could survive to adulthood, they still have phenotypic abnormalities like dramatic weight loss and significant defects in the immune system (Gelling et al., 2008). Therefore, while the global knockout model may be useful for studies focusing on the physiological role of ADAM17, it might not be suitable for investigating its therapeutic role in pathological processes. Moreover, ADAM17-mediated shedding involves a variety of receptors and substrates, which are distributed in different cell types and various tissues. Accordingly, targeting of a specific cell population or tissue becomes a better solution for studies trying to identify ADAM17 and its potential role in certain disease or pathological processes. Since the therapeutic use of pharmacological inhibitors of ADAM17 also has its own limitation, several types of conditional ADAM17 knockout animals have been generated and already expanded our knowledge of ADAM17 roles (Table 1). Using Cre-LoxP technology, these models are amenable for conditional, pre- or post-developmental, deletion of neuronal ADAM17, making them attractive for *in vivo* studies in adult animals devoid of gross anatomical defects. These ADAM17 conditional knockout models should be quite useful for studies focusing on ADAM17-related neurological diseases and neurogenic cardiovascular disorders.

**Table 1:** **Summary of ADAM17 conditional knockout mice**.

**Mouse**	**Targeted cell type and major phenotype**	**References**
ADAM17^flox/flox^-Mx1Cre	This inducible Cre can help create ADAM17 global KO at any time of development; low response to LPS stimulation; low TNFα production; improved energy homestasis	Horiuchi et al., 2007a; Kaneko et al., 2011
ADAM17^flox/flox^-LysMCre	Myeloid cell lineage (monocytes, mature macrophages and granulocytes); reduced proinflammatory cytokines secretion	Horiuchi et al., 2007b
ADAM17^flox/flox^-Sox9Cre	Most tissues except for spleen and thymus; early death, phenotypes are similar to global KO mice	Horiuchi et al., 2009
ADAM17^flox/flox^-Tie2Cre	Endothelial cells; reduced pathological neovascularisation and growth of heterotopically injected tumor; developmental defects in heart valves	Weskamp et al., 2010; Wilson et al., 2013
ADAM17^flox/flox^-HB9Cre	Motor neurons; increased Schwann cell myelination	La Marca et al., 2011
ADAM17^flox/flox^-Foxn1Cre	T cells, no effect on T cell development	Gravano et al., 2010
ADAM17^flox/flox^-Vav1Cre	Hematopoietic cells (and their progenitors); reduced proinflammatory cytokines release; faster neutrophil recruitment	Long et al., 2010; Arndt et al., 2011
ADAM17^flox/flox^-TaglnCre	Vascular smooth muscle cells; no effect on normal vascular development	Weskamp et al., 2010
ADAM17^flox/flox^-Krt14Cre	Keratinocyte cells; normal at birth but develop defects in epidermal barrier soon after birth; develop chronic dermatitis as adults	Franzke et al., 2012

## Conclusion

ADAM17 has more than 70 different substrates, including cytokines, growth factors, receptors, adhesion molecules, and other proteins. There has been substantial progress in understanding how ADAM17 mediates a range of physiological functions and how ADAM17 mediates signaling pathway contributing to the pathological processes in a variety of neurological and cardiovascular disorders. Despite a plethora of data in various fields, the role of ADAM17 in the central regulation of cardiovascular modulation and related cardiovascular diseases is far from being elucidated. Although there has been slow progress in translating the knowledge of ADAM17 into possible new treatments, because of the diversity of its substrates, it remains that ADAM17 could be used as potential novel target for the treatment of cardiovascular diseases. However, this will require treatment strategies with improved efficiency and specificity.

## Author contributions

All authors: JX, SM, CS, AC, JC, CB, VB, EL, and MF, drafted the work, contributed to work design, revised it critically, approved the final version to be published and declare accountable for all aspects of the work.

## Funding

This study was supported by the Conselho Nacional de Desenvolvimento Científico e Tecnológico (CNPq) and by the Coordenação de Aperfeiçoamento de Pessoal de Nível Superior (CAPES), Brazil, Grant 88881.068184/2014-01 to MF, research grants from the American Heart Association (15POST25000010 to JX and 12EIA8030004 to EL) and the National Institutes of Health (HL093178 and GM106392) to EL.

### Conflict of interest statement

The authors declare that the research was conducted in the absence of any commercial or financial relationships that could be construed as a potential conflict of interest.
